# Using mixed effects logistic regression models for complex survey data on malaria rapid diagnostic test results

**DOI:** 10.1186/s12936-018-2604-y

**Published:** 2018-12-05

**Authors:** Chigozie Louisa J. Ugwu, Temesgen T. Zewotir

**Affiliations:** School of Mathematics, Statistics and Computer Science, University of KwaZulu-Natal, Westville Campus, Durban, South Africa

**Keywords:** Generalized Chi-square statistic, Interaction effect, Link function, Odd ratios, Random effects, Sustainable Development Goals (SDGs)

## Abstract

**Background:**

The effect of malaria in Nigeria is still worrisome and has remained a leading public health issue in the country. In 2016, Nigeria was the highest malaria burden country among the 15 countries in sub-Saharan Africa that accounted for the 80% global malaria cases. The purpose of this study is to utilize appropriate statistical models in identifying socio-economic, demographic and geographic risk factors that have influenced malaria transmission in Nigeria, based on malaria rapid diagnostic test survey results. This study contributes towards re-designing intervention strategies to achieve the target of meeting the Sustainable Development Goals 2030 Agenda for total malaria elimination.

**Methods:**

This study adopted the generalized linear mixed models approach which accounts for the complexity of the sample survey design associated with the data. The 2015 Nigeria malaria indicator survey data of children between 6 and 59 months are used in the study.

**Results:**

From the findings of this study, the cluster effect is significant $(P<0.0001)$ which has suggested evidence of heterogeneity among the clusters. It was found that the vulnerability of a child to malaria infection increases as the child advances in age. Other major significant factors were; the presence of anaemia in a child, an area where a child resides (urban or rural), the level of the mother’s education, poverty level, number of household members, sanitation, age of head of household, availability of electricity and the type of material for roofing. Moreover, children from Northern and South-West regions were also found to be at higher risk of malaria disease and re-infection.

**Conclusion:**

Improvement of socio-economic development and quality of life is paramount to achieving malaria free Nigeria. There is a strong link of malaria risk with poverty, under-development and the mother’s educational level.

## Background

Almost half of the world’s population has been at the risk of malaria, but in terms of mortality and morbidity attributed to the disease, the African children aged under 5 years have been mostly affected. According to the World Health Organization (WHO), year 2016 alone recorded 216 million cases of malaria infection and 445,000 mortality cases worldwide, of which 91% occurred in African countries [1].

Of the fifteen countries in sub-Saharan Africa that accounted for 91% of the global malaria cases, Nigeria bears the major burden of about 40% which included 25% infant mortality, close to 31% under-five mortality and nearly 11% maternal mortality in annual bases [2]. Similarly, there are more than 100 million clinically diagnosed malaria cases, of which approximately 300,000 malaria associated childhood deaths occur yearly in Nigeria [3]. The effect of malaria disease in Nigeria is worrisome and has remained a leading public health issue in the country, hence, a major cause of about 60% unscheduled hospital visits and more than 30% hospitalization of children and pregnant women in Nigeria [4]. Malaria parasitaemia is mainly observed during the first pregnancy, but decreases afterwards; pregnancy in turn reduces the inhabitance of normal immune response due to the infection and as such, may cause severe cases among them [5]. Malaria infection of the mother increases the risk of abortion, stillbirth and also the odd of congenital malaria transmission to newborns which will eventually reduce the infant’s survival chances.

In Nigeria, malaria is endemic and has contributed to the huge economic loss to the nation due to its negative impact in the capacity of a debilitating work force and draining national resource due to the disease control and treatment [6]. Moreover, malaria disease affects mostly agricultural regions, the infection weakens its victim’s strength by making the individual succumb to other infectious diseases and as such, affects country’s agricultural efficiency [7].

The Nigerian government, through the National Control Programme (NMCP), together with several non-governmental partners such as Roll Back Malaria (RBM) have made and are still making drastic efforts in reducing malaria transmission and associated child death through the implementation of (2009–2013) malaria control strategic plan and on the wide dissemination of malaria knowledge through mass distribution of long-lasting insecticide-impregnated nets (LLINs) within the selected state of the country. Their effort yielded a huge result within 2010–2015 by reducing malaria prevalence from 52 to 45% [2]. The NMIS outcome between 2010–2015 indicated an improvement of about 5% in malaria prevalence reduction, though some regions are still lagging behind with tremendous malaria cases [2]. It has been and still being a leading cause of death among children between the age bracket (6 months–5 years) in Nigeria, mostly among the poor and rural communities [1, 2].

Recent research on malaria prevalence in other malaria endemic countries [8–14] and in Nigeria [5–7, 15–21] have identified major factors such as unavailability of LLINs, presence of other infections, illiteracy on the part of parents or caregiver, poverty, and inadequate dissemination of malaria knowledge, to be highly associated with the malaria disease transmissions.

Most of the studies in Nigeria have been largely limited to community and hospital based simple random sample survey among pregnant mothers [5, 17–19], however, very few studied clinical malaria cases among children [15, 20, 22, 23]. Using data from 2010 Nigeria malaria indicator survey and the mapping malaria risk dataset in Africa (MARA), [5, 24] employed standard logistic regression and a Bayesian geostatistical modeling. Their results showed that environmental and climatic factors are major predictors of malaria parasite infection. Also, [25] used the 2008, Nigeria demographic health survey data (NDHS) to study the relationship between children’s fever report and poverty in Nigeria. This study found that low fever occurrences were reported in the households that posses mosquito bed nets. However, no studies have been done on under-five malaria risk indicators in Nigeria using National level data.

The world is presently in the post MDG era and recently the WHO Global Technical Strategy for malaria 2016–2030 is endorsed with the objective of drastically reducing global malaria occurrences by at least 90%, malaria related death by at least 90%, eradicating malaria in at least 35 countries and preventing re-emergence of malaria in all the malaria free countries [26]. To meet the SDGs 2030 target on total malaria elimination and to also achieve Nigeria’s own 2014–2020 agenda in reducing malaria—related deaths to zero level, investigation into individual and household (socio-economic, geographic, demographic and environmental) determinants of malaria prevalence and associated child mortality is paramount for the best strategic interventions. In other to achieve great success in re-strategizing policy measures, policy implementation that will extensively lower the malaria burden in the country, consistent investigation into the epidemiology and the major risk factors associated with malaria infection is paramount [5, 15, 24].

In this paper, the 2015, Nigeria malaria indicator survey data (NMIS) was utilized to investigate the factors associated with malaria RDT status of children aged under 5 years in Nigeria and hence, this study contribute to highlight measures that may be implemented towards re-designing intervention strategies to achieve the SDGs 2016–2030 Agenda for total malaria elimination in Nigeria.

## Methods

### The data

The 2015 Nigeria Malaria Indicator Survey (NMIS) has been conducted by the National Population Commission (NPopC), the National Bureau of Statistics (NBS), the Malaria Elimination Program (NMEP) and the Malaria Partnerships in Nigeria, which was supported by PMSI-USAID, GFATM, DFID, UNICEF, WHO and the United Kingdom Department for International Development (DFID) and was carried out from October through December 2015 [2]. This was the second and more comprehensive malaria indicator survey implemented just one year after the first survey in 2010 and also after one year in the development of the new national malaria strategic plan that covers 2014–2020 [2]. This is an internationally recognized household survey, which is periodically conducted in high malaria endemic countries at the time of malaria season for the purpose of providing national level information on malaria indicators and prevalence. The NMIS captured a number of individual and household characteristics. A sample of 8148 households was selected from 333 clusters across the country, of which 138 clusters are in urban areas and about 195 clusters are from rural areas [2].

Children aged 6–59 months, who were born from women in the 8148 sampled households were tested for malaria and anaemia using blood samples. A total of 5236 children participated in the 2015 NMIS. Hence, children aged 6–59 months were used in this study.

### Response variable

Malaria rapid diagnostic tests (RDTs) are immuno-chromatography form of tests which detect the presence of malaria antigens discharged from the parasitized red blood cells.

The World Health Organization (WHO) has supported the use of both microscopy and rapid diagnostic testing approach for malaria diagnosis. Microscopy being the oldest has been recognized as the standard approach for malaria diagnosis, but the application is however tedious. Microscopy requires an experienced (laboratory specialist) microscopist, relaxed environment, time, degree of operational expertise and cost [27]. In remote rural communities, microscopy may be subject to false negative results due to the fact that, malaria results are highly subject to human error attributed to loss of parasite during the staining procedure. Conversely RDTs does not require specialized equipments, long process and skilled personnel. The recent development in introduction of RDTs has been so fruitful towards early detection, prompt treatment and reduction of severe cases for effective ’test and treat-strategy recommended by WHO [28]. The RDT method has gained popularity in every situation and has been mostly applied during population based survey for immediate intervention, because it gives rapid result in a space of 15–30 min [29, 30]. Moreover, the systematic reviews have proven that the RDTs approach is a reliable diagnosis for malaria infection [31, 32].

Therefore, for the purpose of this study, the dependent variable is the binary response from the children RDT outcome where 1 signifies the presence of malaria infection and 0 for no malaria infection.

### Explanatory variables

The explanatory variables were selected to give an answer to the study objective. The selected variables were based on previous studies to critically compare results. These include;

i. Child’s characteristics: sex of child (female, male); age of child (6–59 months; the anemic status of a child (yes, no); child treated fever before malaria test (yes, no).
ii. Geospatial: sampling enumeration clusters; region (North central, North East, North West, South East, South South, South West); type of place of residence (rural, urban).
iii. Mother’s characteristics: mother’s educational level (no education, primary, secondary and higher education)
iv. Head of household’s characteristics: age of head of household (continuous), gender of head of household (female, male).
v. Socio-economic characteristics of the household: wealth index (poorer, middle-range, richer, richest); number of household members (continuous); availability of some critical household possessions such as radio (yes, no); television (yes, no); electricity (yes, no); household wall material (mud-wood-others), roof (thatched-wood-others, zinc-metal-roof), main floor (cement-wood-other, palm-sand-others); source of drinking water (protected water, tap-piped water, unprotected water).
vi. Environmental and sanitation characteristics: Use of mosquito indoor residual spray (yes, no); use of mosquito net (yes, no); total number of nets used (continuous); toilet facility (flush toilet, no toilet, pit-latrine); distance from water source (< 30 min, 31–49 min, 50–90 min, > 90 min, on premises).

## Statistical methods

Under complex survey design with unequal weighting, the ordinary logistic regression statistical estimates will be inappropriate for the analysis [33–36]. Accordingly, this study employed the mixed effects logistic regression model approach under the generalized linear mixed models (GLMMs) framework which accounts for the complexity of the sampling design. Moreover, the GLMM accommodates both random and fixed effects in the model [37–39].

Let $y_{ikt}$ be the binary response variable of the *i*th child in the *k*th household within the *t*th sampling clusters. Let $\pi _{ikt}=P(y_{ikt}=1)$ denote the probability that an *i*th child RDT outcome in the *k*th household, within the *t*th cluster is positive. Suppose $\mathbf{x }^{\prime }_{ikt}$ is the row vector of covariates, which corresponds to the *i*th child in the *k*th household and the *t*th cluster and $\beta$ is the vector of unknown model parameters. Then, following [14, 40–42], the generalized linear mixed models (GLMMs) framework of the mixed effect logistic regression models formulates the logit of $\pi _{ikt}$ as a function of the covariates $\mathbf{x }^{\prime }_{ikt}$ and the random cluster effect $\gamma _{t}$, as:

$$logit(\pi _{ikt})= log \left [\frac{\pi _{ikt}}{1-\pi _{ikt}}\right]=\mathbf{x }^{\prime }_{ikt}\beta + \gamma _{t}.$$

## Results

Weighted mixed effects logistic regression model was regressed on the explanatory variables. The weights were the sampling weights which were used in the NMIS complex survey design. To avert the influence of confounding variables, all the main effects were retained in the model. It was assessed as to whether any interaction terms were needed to be incorporated into the model. This was examined by fitting each of the two-way interaction terms formed from all the explanatory variables, one at a time to the model that had all the main effects. Interactions which highly improved the goodness of fit and highly significant (P < 0.10) were sequentially added to the model until there was no significant interaction effect to be included in the model.

Accordingly, only four interaction effects, namely region and type of place of residence, wealth index and type of place of residence, age and gender of the head of household, and age of head of the household and the number of household members. Consequently, the final model included all the main effects and the four two-way interaction effects.

**Table 1:** Covariance parameter estimates

Cov. parameter	Estimate	Standard error	Z value	P-value
Clusters	0.8853	0.1142	7.76	< 0.0001
Residual	0.9022	0.0183	49.42	< 0.0001

All the model fits and estimates were obtained using the SAS GLIMMIX procedure [43]. The model fit was assessed using the ratio of the generalized chi-square statistics and it’s degree of freedom, which yielded 0.90. This result indicated a good model fit with no residual over-dispersion. The random effect cluster, which accounted for the complexity of the sampling design is significant as shown in Table 1. The result shows that there is heterogeneity between clusters. The cluster variability accounts about 50% the total variability of under-five child RDT outcome.

**Table 2:** Type III tests for fixed effects

Effects	Num DF	Den DF	F value	P-value
Region	5	314	6.70	< 0.0001
Mother’s education	3	494	4.97	0.0021
Child’s age	4	1151	53.14	< 0.0001
Child’s anaemia status	1	305	185.47	< 0.0001
Age of household head	1	4868	4.59	0.0322
Toilet facility	2	285	12.40	< 0.0001
Number of household members	1	4868	8.04	0.0046
Cluster altitude in metres	1	71	0.63	< 0.0001
Availability of electricity	1	61	0.79	0.0457
Sex of a child	1	320	1.26	0.2627
Prior child’s fever status	1	4868	89.96	< 0.0001
Distance from water source	3	354	0.19	0.9000
Mosquito indoor spray	1	30	0.12	0.7292
Number of mosquito nets used	1	4868	0.28	0.5967
Child slept under bed net	1	285	0.18	0.6683
Number of rooms	1	4868	0.22	0.6423
Household’s main floor	2	331	1.84	0.1610
Households’s main wall	2	228	1.44	0.2395
Household’s main roof	2	230	6.40	0.0012
Households’s wealth index	4	466	1.74	0.1430
Availability of television	1	190	0.05	0.8302
Gender of head of household	1	148	0.67	0.4132
Place of residence	1	314	13.55	0.0003
Source of drinking water	2	255	1.87	0.1555
Number of household members * age of head of household	1	4868	5.09	0.0241
Gender * age of head of household	1	4868	2.76	0.0967
Wealth index * type of residence	4	466	2.49	0.0424
Region * type of place of residence	5	314	1.95	0.0862

The type III tests for the fixed effects in Table 2 shows that region, mother’s level of education, child’s anaemia level, age of the child, age of head of household, toilet facility, number of household members, cluster altitude in meters, availability of electricity, type of place of residence (urban or rural) and child’s fever report two weeks prior to survey and the interactions between number of household members and age of head of household, gender and age of head of household, region and type of place of residence significantly associated child’s malaria RDT outcome.

**Table 3:** Parameter estimates of odds ratio for the main effects

Effect	Estimates	Standard error	Odd ratio	95% CI		P-value
				Lower	Upper	
Intercept	− 4.8896	0.5943	0.0075	0.0023	0.0241	< 0.0001
Region (Ref. North West)						
South East	0.1086	0.3779	1.1147	0.5315	2.3380	0.7741
South South	− 0.5394	0.4571	0.5831	0.2380	1.4283	0.2389
South West	0.3979	0.3578	1.4887	0.7383	3.0017	0.2670
North Central	− 0.3953	0.4453	0.6735	0.2814	1.6120	0.3753
North East	− 1.1844	0.4844	0.3059	0.1184	0.7906	0.0150
Place of residence (Ref. urban)						
Rural	1.5215	0.4590	4.5791	1.8624	11.2587	0.0010
Household wealth index (Ref. richest)						
Poorest	1.2669	0.4368	3.5498	1.5080	8.3564	0.00391
Poorer	1.7230	0.3731	5.6013	2.6959	11.6380	< 0.0001
Middle-range	0.8989	0.2645	2.4569	1.4630	4.1260	0.0007
Richer	0.6020	0.1953	1.8258	1.2451	2.6772	0.0022
Mother’s educational level (Ref. more than secondary)						
No education	0.7156	0.2077	2.0454	1.3614	3.0731	0.0006
Primary	0.4260	0.2034	1.5311	1.0277	2.2811	0.0368
Secondary	0.4363	0.1868	1.5470	1.0727	2.2310	0.0199
Anti-malaria spraying (Ref. yes)						
No	− 0.1229	0.3516	0.8844	0.5020	1.7616	0.7292
Use of mosquito nets (Ref. yes)						
No	− 0.0348	0.0811	0.9658	0.8238	1.1323	0.6683
Child’s age in months (Ref. age 6–12 months), months						
Age 13–24	0.5329	0.1220	1.7039	1.3415	2.1641	< 0.0001
Age 25–36	0.9648	0.1224	2.6243	2.0645	3.3358	< 0.0001
Age 37–48	1.2785	0.1218	3.5912	2.8286	4.5596	< 0.0001
Age 49–59	1.6047	0.1259	4.9764	3.8882	6.3883	< 0.0001
Child’s gender (Ref. male)						
Female	− 0.0756	0.0673	0.9272	0.8126	1.0544	0.2627
Child’s anaemic status (Ref. not anaemic)						
Anemic	1.0928	0.0802	2.9826	2.5486	3.4906	< 0.0001
Prior child’s fever status (Ref. no fever)						
Child had fever	0.6767	0.0714	1.9674	1.7106	2.2627	< 0.0001
Age of head of household (continuous)	0.0189	0.0059	1.0191	1.0074	1.0309	0.0013
Number of household members (continuous)	0.0961	0.0339	1.1008	1.0301	1.1762	0.0046
Number of net used	− 0.0141	0.0266	0.9860	0.9359	1.0388	0.5967
Number of rooms (continuous)	− 0.0119	0.0257	0.9881	0.9396	1.0643	0.6423
Cluster altitude in metres (continuous)	0.0003	0.0023	1.0003	0.9910	1.1003	< 0.0001
Availability of electricity (Ref. yes)						
No	0.1372	0.1306	1.1471	0.8880	1.4817	0.0457
Availability of television (Ref. yes)						
No	0.0261	0.1216	1.0265	0.8088	1.3027	0.8302
Type of toilet facility (Ref. flush toilet)						
No toilet facility	0.4919	0.1540	1.6354	1.2093	2.2117	0.0016
Pit latrine	− 0.0582	0.1387	0.9434	0.7189	1.2382	0.6749
Distance from water source to the household (Ref. > 90 min), min						
< 30	− 0.0338	0.0857	0.9668	0.8174	1.1437	0.6937
31–49	− 0.0006	0.2413	0.9995	0.6228	1.6038	0.9982
50–90	0.1116	0.2148	1.1181	0.7339	1.7034	0.6036
Household main roof (Ref. zinc/metal)						
Wood material	− 0.1845	0.1285	0.8315	0.6464	1.0697	0.1524
Thatched/palm leaf	− 0.3325	0.1273	0.7171	0.5588	0.9204	0.0096
Household main floor (Ref. wood material)						
Cement	0.0443	0.1387	1.0453	0.7965	1.3718	0.7497
Localdung plaster/earth	0.2404	0.1594	1.2718	0.9305	1.7382	0.1326
Household main wall (Ref. mud/bamboo/wood)						
Cement block	0.0894	0.1146	1.0936	0.8736	1.3690	0.4361
No walls	− 0.2099	0.1568	0.8107	0.5962	1.1023	0.1821
Type of drinking water (Ref. unprotected water)						
Protected water	− 0.1229	0.1626	0.8844	0.6430	1.2163	0.4505
Tap/pipped water	− 0.1962	0.1017	0.8218	0.6733	1.0031	0.0549

In this study, the results of the main effect parameter estimates, the odds ratios (OR), the 95% confidence intervals and the P-values are shown in Table 3. Highlighted also were some of the results from Table 3.

The age effect shows that as a child gets older, the odd of malaria RDT positive outcome. The risk of anaemia was found to be associated with malaria status of under-five children. The odds of positive RDT outcome for under-five anaemic children is 3.16 times more than that of the non-anaemic, but otherwise identical children.

The mother’s educational level was significantly associated with the risk of malaria. The positive outcome of the malaria RDT increased with a decreasing level of the mother’s education. A child who has an illiterate mother is $2.0454\,(P\text{-}value = 0.0006)$ more likely to have malaria positive RDT outcome otherwise identical mother with a higher educational level.

**Table 4:** Parameter estimates of odds ratio for the interaction effects

Parameter	Estimates	Standard error	Odd ratio	95% CI		P-value
				Lower	Upper	
Wealth index * type of place of residence (Ref. richest and urban)						
Poorest * rural	− 1.0308	0.5076	0.3567	0.1319	0.9647	0.0429
Poorer * rural	− 1.2839	0.4490	0.2770	0.1149	0.6677	0.0044
Middle-range * rural	− 0.8036	0.3632	0.4477	0.2197	0.9124	0.0274
Richer * rural	− 0.8738	0.3237	0.4174	0.2213	0.7871	0.0072
Number of household members * age of household head	− 0.0015	0.0007	0.9984	0.9972	0.9998	0.0241
Gender * age of head of household						
Female	− 0.0122	0.0073	0.9879	0.9738	1.0022	0.0967
Region * type of place of residence (Ref. North West and urban)						
South East * rural	− 0.9842	0.5224	0.3737	0.1342	1.0405	0.0605
South South * rural	− 0.35805	0.5361	0.6991	0.2444	1.9992	0.5048
South West * rural	0.2646	0.5000	1.3028	0.4889	3.4712	0.5972
North Central * rural	0.1137	0.5056	1.1204	0.4159	3.0182	0.8222
North East * rural	0.7067	0.5449	2.0273	0.6968	5.8985	0.1956

**Fig. 1:**
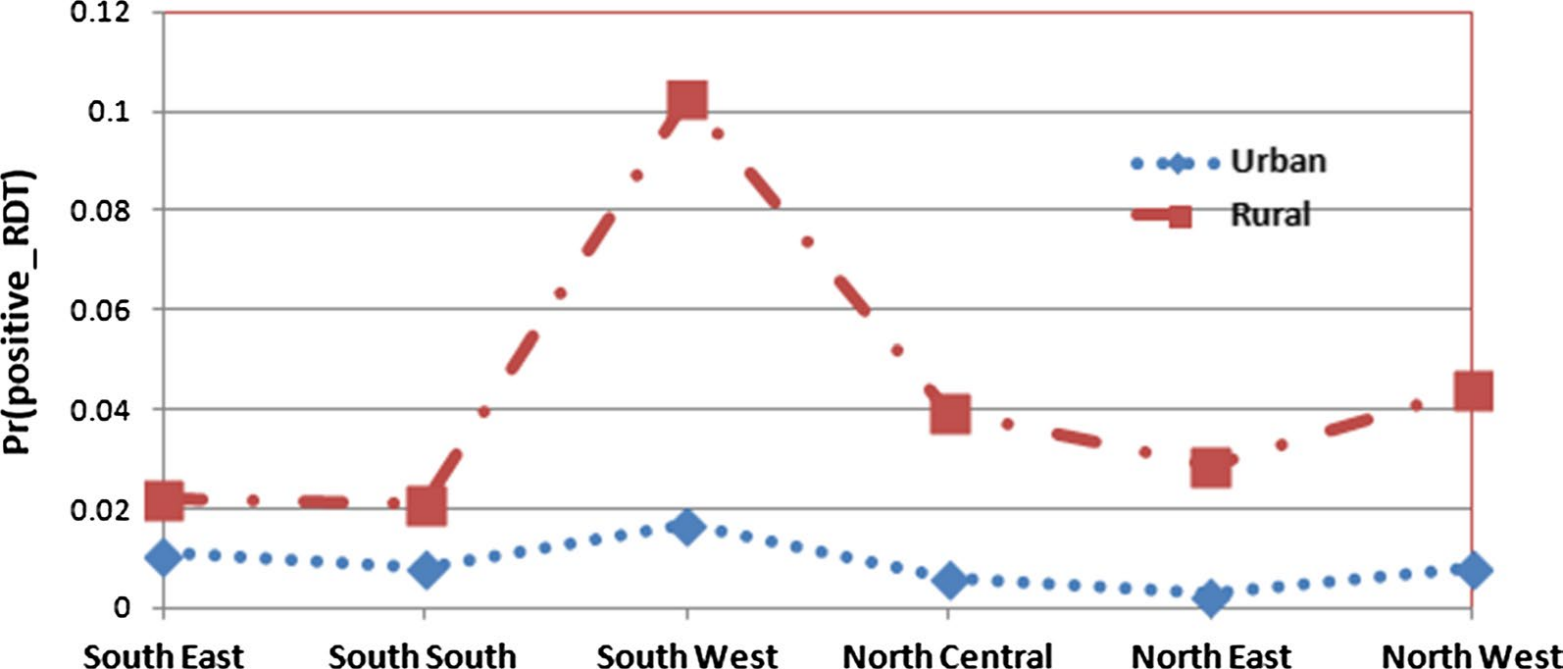
Predicted probabilities for positive malaria RDT by region and type of place of residence with 95% confidence limits

The interaction effects estimate summary is given in Table 4. The interaction effects between regions (South East, South South, South West, North Central, North East and North West) and type of place of residence (urban or rural) is presented in Fig. 1. Figure 1 shows that malaria prevalence is higher in rural areas than that of the urban areas in all the regions of Nigeria.

**Fig. 2:**
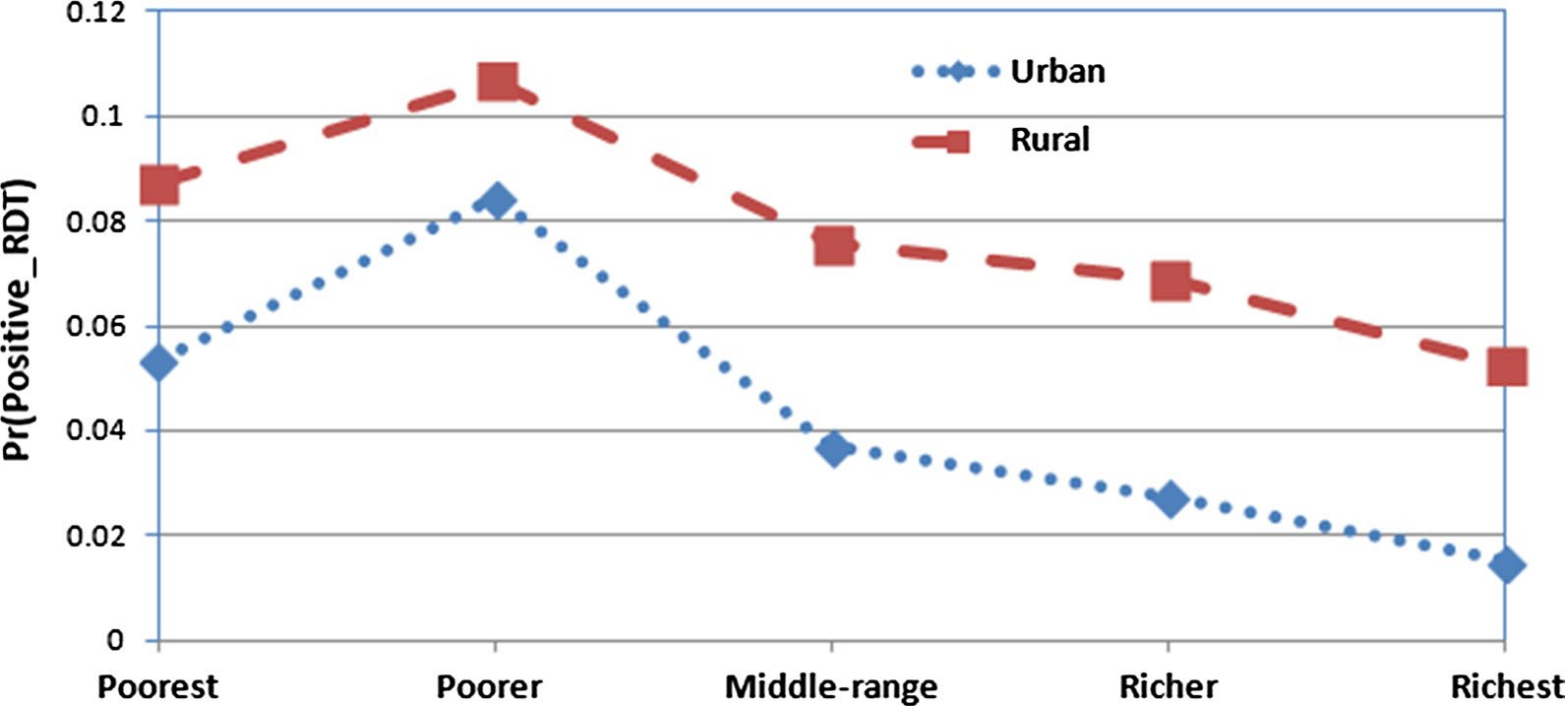
Predicted probabilities for positive malaria RDT by household wealth index and type of place of residence with 95% confidence limits

Figure 2 presents the interaction effect involving type of place of residence and wealth index (socio-economic status) of households. The prevalence of malaria was significantly very high among poorer and poorest in both urban and rural households compared to (middle-range, richer and richest) households.

**Fig. 3:**
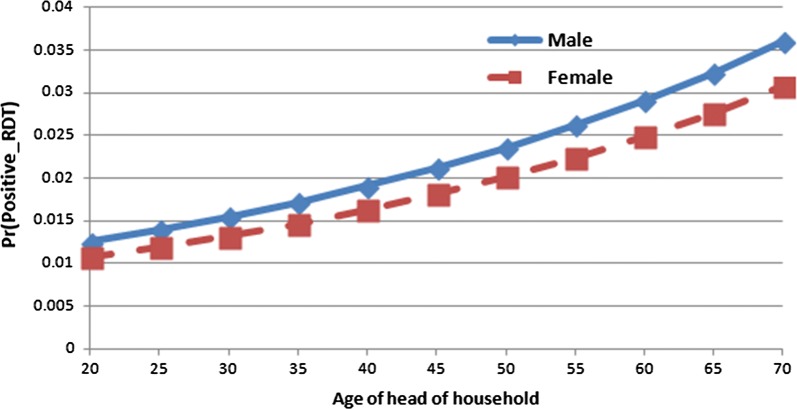
Predicted probabilities for positive malaria RDT by gender and age of head of household with 95% confidence limits

The interaction between gender and age of head of household is presented in Fig. 3. The figure shows that increase in ages of both male and female head of household increases the odd of malaria prevalence on the under-five children.

**Fig. 4:**
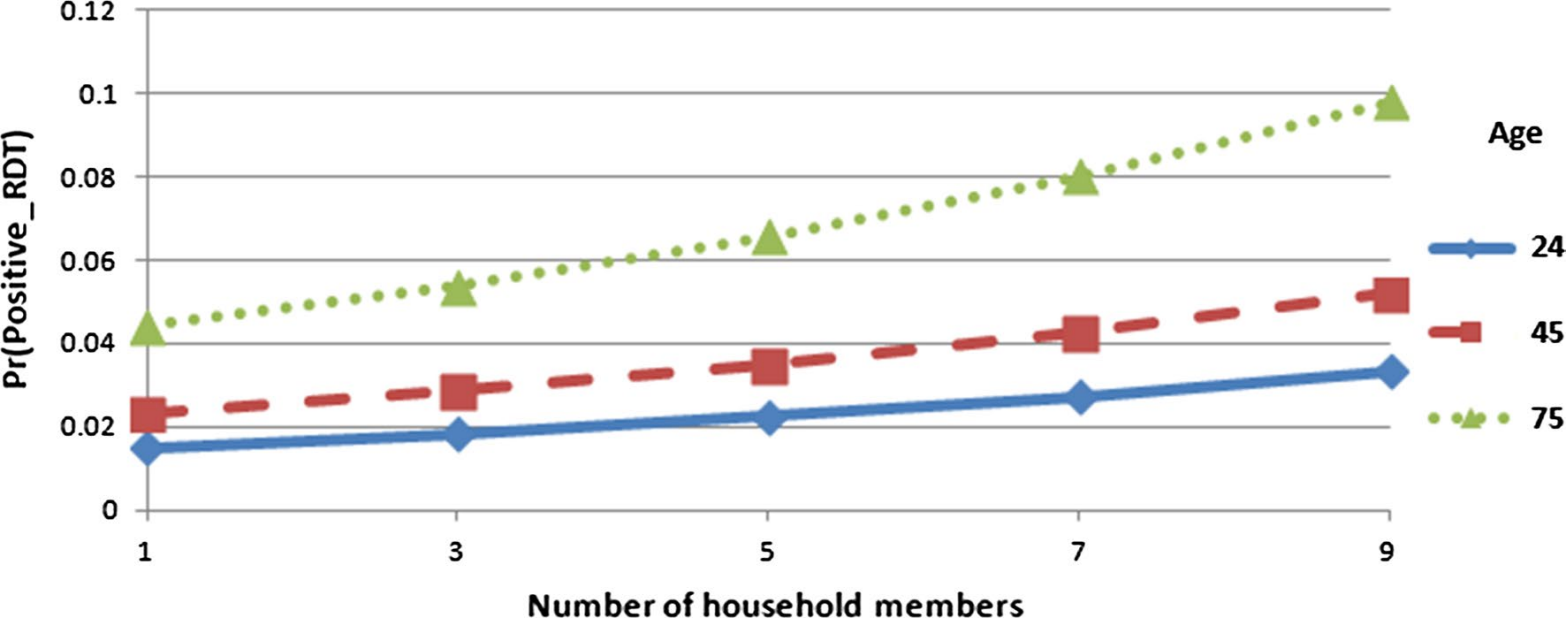
Predicted probabilities for positive malaria RDT by age of head of household and number of household members with 95% confidence limits

Finally Fig. 4 presents the interaction effect between the ages of head of household and household size. In Fig. 4, it shows that the number of household members increases as the age of head of household increases which also impact heavily on the malaria RDT outcome of children under-5 years in Nigeria.

## Discussion

Understanding the critical risk factors and prevalence of malaria among children in a household is very informative and crucially important in re-designing appropriate intervention strategies for final malaria eradication in Nigeria. This study is aimed to investigate the determinant of malaria infection among Nigerian children aged under 5 years using the 2015 NMIS data.

The use of mosquito bed net, has insignificant effect on the under-five child’s malaria RDT outcome. This result is in line with the findings of [9, 14, 44]. But this is contrary to the results obtained from studies in Ethiopia [8], Burkina-Faso [13] and Rwanda [45], they observed significant relationship between those predictor variables and malaria prevalence among children under 5 years. The Roll Back Malaria Partners, the WHO and many other private donors have contributed tremendously in mosquito bed net distribution in many regions in Nigeria, which might contribute to the reason for the insignificant effect of mosquito bed net on under-five children RDT outcome.

In this study, it was observed that as a child gets older, the odd of malaria infection increases. Children between the ages of 6–24 months are found to be less affected by the malaria parasite than older children between ages 49–59 months. This result is consistent with recent results found by many studies on under-5 year children. From the findings, it was observed that a child’s vulnerability to malaria infection increases with increase in age, older children being more at the risk of malaria infection [9, 14]. This was evident from recent studies on under-five children that malaria positivity increasing with age [4, 8, 12, 16, 17]. A child between age 0 and 13 months might still be protected by the maternal antibodies, mothers give more attention to children under one year and as the child gets older outdoor activities exposes them to more malaria risk [9, 15, 16]. Similarly, the results has shown that a child’s gender has no association with malaria infection, which is similar to the results obtained by [8, 13, 14].

This study has observed a similar result with [25] that, malaria RDT status of the under-five children in Nigeria was positively associated with anaemia risk. This means that for the anaemic children, the RDT outcome tends to be positive and may require further investigation to ascertain if the result might be a case of RDT sensitivity issues.

Maternal education plays a very important role in the child’s health in a household. The result of this study shows a significant association between educational level of the child’s mother and malaria prevalence. This finding is similar to the studies carried out by [10, 14]. It is believed that since mothers are at the centre of family well being, their exposure through education is paramount to understanding health related issues and preventive measures for malaria infections towards their children.

Regarding geographical impact on malaria prevalence, the finding shows a significant geographical variation in malaria prevalence among Nigerian children. Children living in the North West, North Central, North East, and South West were highly associated with high malaria risk compared to those residing in the South East and South West regions. This result is consistent with similar results found from published studies [4, 24].

## Conclusion

In this paper, a GLMM was fitted and the complexity of the designs were incorporated in the model. The heterogeneity among clusters is found to be significant and the effects were accounted in the analysis of the factors effect.

The level of under-development in Nigeria presents a serious challenge for malaria eradication. The findings from this study have also provided insight into socio-economic and mother’s educational level. Mother’s educational level has been found to influence her children’s vulnerability to malaria infection. Having better educated mothers is a human capital for the nation and the family at large. Therefore, child malaria eradication and information strategy should incorporate mother’s education enhancement.

Investigation into the significant association between under-five children RDT outcome and their anaemic test will be one of the alarming results about RDT diagnostic method. This is perhaps that anaemic children RDT outcome tends to show positive outcome or vice-versa. Therefore, one of the future direction of this research is to investigate the joint distribution of anaemia test status and the RDT outcome on under-five children.

## Abbreviations

OR: Odds ratios
EAs: Enumeration area
PSU: Primary sampling unit
GLMMs: Generalized linear mixed models
RDT: Rapid diagnostic test
WHO: World Health Organization
MDGs: Millennium Development Goals
SDGs: Sustainable Development Goals
DFID: Department for International development
NPopC: National Population Commission
NPHC: National Population and housing Census
UNICEF: The United Children’s Emergency Fund
USAID: The United States Agency for International Development
GFATM: Global Fund to Fight AIDS, Tuberculosis and Malaria
NMEP: National Malaria Eradication Program
